# 1-(2-Bromo-2-de­oxy-β-d-xylofuranos­yl)uracil

**DOI:** 10.1107/S1600536810051081

**Published:** 2010-12-11

**Authors:** Zhong-Gao Zhou, Shun-Guo Fu, Wu-Leng Lai, Chun-Feng Wang

**Affiliations:** a College of Chemistry and Life Science, Gannan Normal University, Ganzhou 341000, People’s Republic of China; b School of Chemical and Environmental Sciences, Henan Normal University, Xinxiang 453007, People’s Republic of China

## Abstract

In the title compound, C_9_H_11_BrN_2_O_5_, the ribofuran­ose ring has a C2-*exo*, C3-*endo* twist configuration and is attached to the uracil unit *via* a β-N_1_-glycosidic bond. The crystal structure is stabilized by two inter­molecular O—H⋯O inter­actions and one inter­molecular N—H⋯O inter­action.

## Related literature

For the synthesis of the title compound and its analogues, see: Shakya *et al.* (2010 ▶). For a related structure, see: Suck *et al.* (1972 ▶). For the use of the title compound as a pharmaceutical inter­mediate, see: Haraguchi *et al.* (1993 ▶); Kittaka *et al.* (1992 ▶); Pozharskii *et al.* (1997) ▶; Sairam *et al.* (2003 ▶). For the biological activity of nucleoside derivatives, see: Johar *et al.* (2005 ▶).
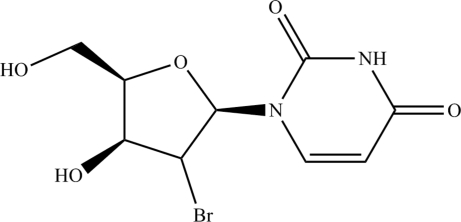


## Experimental

### 

#### Crystal data

C_9_H_11_BrN_2_O_5_
*M* *_r_* = 307.11
Orthorhombic, 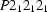

*a* = 4.8444 (3) Å
*b* = 12.7237 (10) Å
*c* = 17.4388 (13) Å
*V* = 1074.90 (13) Å^3^
*Z* = 4
Mo *K*α radiation
μ = 3.84 mm^−1^
*T* = 296 K
0.30 × 0.20 × 0.06 mm

#### Data collection

Bruker SMART CCD area-detector diffractometer
Absorption correction: multi-scan (*SADABS*; Bruker, 2008 ▶) *T*_min_ = 0.583, *T*_max_ = 0.746
6683 measured reflections
2091 independent reflections
1956 reflections with *I* > 2σ(*I*)
*R*_int_ = 0.023

#### Refinement

*R*[*F* ^2^ > 2σ(*F* ^2^)] = 0.021
*wR*(*F* ^2^) = 0.048
*S* = 1.02
2091 reflections
155 parameters
H-atom parameters constrained
Δρ_max_ = 0.20 e Å^−3^
Δρ_min_ = −0.34 e Å^−3^
Absolute structure: Flack (1983 ▶), 834 Friedel pairs
Flack parameter: 0.016 (9)

### 

Data collection: *SMART* (Bruker, 2008 ▶); cell refinement: *SAINT* (Bruker, 2008 ▶); data reduction: *SAINT*; program(s) used to solve structure: *SHELXS97* (Sheldrick, 2008 ▶); program(s) used to refine structure: *SHELXL97* (Sheldrick, 2008 ▶); molecular graphics: *SHELXTL* (Sheldrick, 2008 ▶) and *DIAMOND* (Brandenburg, 1999 ▶); software used to prepare material for publication: *SHELXTL*.

## Supplementary Material

Crystal structure: contains datablocks I, global. DOI: 10.1107/S1600536810051081/hg2764sup1.cif

Structure factors: contains datablocks I. DOI: 10.1107/S1600536810051081/hg2764Isup2.hkl

Additional supplementary materials:  crystallographic information; 3D view; checkCIF report

## Figures and Tables

**Table 1:** Hydrogen-bond geometry (Å, °)

*D*—H⋯*A*	*D*—H	H⋯*A*	*D*⋯*A*	*D*—H⋯*A*
O2—H2*B*⋯O4^i^	0.82	2.03	2.841 (2)	169
N2—H2*C*⋯O3^ii^	0.86	2.17	2.983 (2)	158
O3—H3*B*⋯O5^iii^	0.82	1.96	2.769 (2)	167

Symmetry codes: (i) 
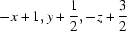
; (ii) 
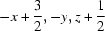
; (iii) 
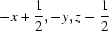
.

**Fig. 1.**
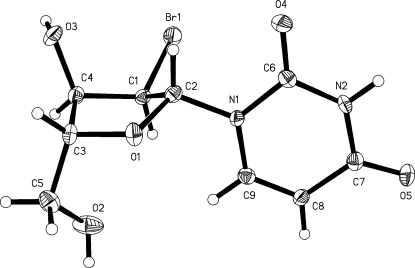
The molecular structure of the title compound, showing the atom-numbering scheme. Displacement ellipsoids are drawn at the 30% probability level.

**Fig. 2.**
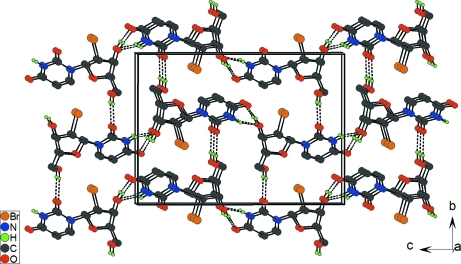
The three-dimensional structure of the title compound formed by intermolecular hydrogen bonds viewed down the a axis. The intermolecular hydrogen bonds are shown as dashed lines.

## supplementary crystallographic information

### Comment

In the last few decades, there has been dramatic progress in the synthesis of the nucleoside analogues for their biological evaluation of the anticancer activity (Johar *et al.*, 2005; Shakya *et al.*, 2010; Suck *et al.*, 1972). The title compound (I) can be used as important pharmaceutical intermediates (Haraguchi *et al.*, 1993; Kittaka *et al.*, 1992; Pozharskii *et al.*, 1997; Sairam *et al.*, 2003). The synthetic procedure is described below. To know the relative stereochemistry of the anomeric position in the ribofuranose ring, it is necessary to gain the well defined structure of (I) by X-diffraction method. The molecular structure of the title compound is shown in Fig. 1. From the single-crystal structure we observed that the ribofuranose ring has a C2-*exo*, C3-*endo* twist configuration and the anomeric carbons are always β configuration in the crystal packing. The crystal structure of (I) is stabilized by two intermolecular O—H···O interactions and one intermolecular N—H···O interaction (Table 1, Fig. 2).

### Experimental

All reagents and solvents were used as obtained commercially without further purification. NMR spectra was recorded on Bruker AV 400 MHz NMR spectrometers at ambient temperature. The title compound was prepared according to the reported procedure (Shakya *et al.*, 2010). Detritylation of 1-(3-Bromo-3-deoxy-5-O-trityl-β-D-arabinofuranosyl)uracil using 80% aqueous acetic acid (*v*/*v*) at 90 °C for 30 min, then cooled to room temperature, after the solvent were distilled off a white solid of the title compound was obtained in about 70% yield. 1H NMR (400 MHz, DMSO-*d6*): *δ* 3.65–3.77 (m, 2H, H-5'), 4.26–4.39 (m, 3H, H-2', H-3', H-4'), 4.89 (t, *J* = 4.88 Hz, 1H, 5'-OH), 5.64 (dd, *J* = 8.54 and 1.83 Hz, 1H, H-5), 6.04 (d, *J* = 3.05 Hz, 1H, H-1'), 6.09 (d, *J* = 1.83 Hz, 1H, 3'-OH), 7.72 (d, *J* = 7.93 Hz, 1H, H-6), 11.39 (s, 1H, NH). In a sample vial, colorless block-shaped single crystals were grown from DMSO and water (*v*/*v* = 1:1) at room temperature.

### Refinement

The N-bound and the C-bound H atoms were positioned geometrically and refined using a riding model: N—H = 0.86 Å and C—H = 0.93–0.98 Å, with *U*_iso_(H) = 1.2*U*_iso_(N,*C*); while the O-bound H atoms were placed in idealized positions and constrained to ride on their parent atoms: O—H = 0.82 Å, with *U*_iso_(H) = 1.5 times *U*_iso_(O).

### Crystal data

**Table:**

C_9_H_11_BrN_2_O_5_	*F*(000) = 616
*M**_r_* = 307.11	*D*_x_ = 1.898 Mg m^−^^3^
Orthorhombic, *P*2_1_2_1_2_1_	Mo *K*α radiation, λ = 0.71073 Å
Hall symbol: P 2ac 2ab	Cell parameters from 3827 reflections
*a* = 4.8444 (3) Å	θ = 2.3–26.6°
*b* = 12.7237 (10) Å	µ = 3.84 mm^−^^1^
*c* = 17.4388 (13) Å	*T* = 296 K
*V* = 1074.90 (13) Å^3^	Block, colourless
*Z* = 4	0.30 × 0.20 × 0.06 mm

### Data collection

**Table:**

Bruker SMART CCD area-detector diffractometer	2091 independent reflections
Radiation source: fine-focus sealed tube	1956 reflections with *I* > 2σ(*I*)
graphite	*R*_int_ = 0.023
phi and ω scans	θ_max_ = 26.0°, θ_min_ = 2.0°
Absorption correction: multi-scan (*SADABS*; Bruker, 2008)	*h* = −5→5
*T*_min_ = 0.583, *T*_max_ = 0.746	*k* = −12→15
6683 measured reflections	*l* = −17→21

### Refinement

**Table:**

Refinement on *F*^2^	Secondary atom site location: difference Fourier map
Least-squares matrix: full	Hydrogen site location: inferred from neighbouring sites
*R*[*F*^2^ > 2σ(*F*^2^)] = 0.021	H-atom parameters constrained
*wR*(*F*^2^) = 0.048	*w* = 1/[σ^2^(*F*_o_^2^) + (0.0196*P*)^2^] where *P* = (*F*_o_^2^ + 2*F*_c_^2^)/3
*S* = 1.02	(Δ/σ)_max_ = 0.001
2091 reflections	Δρ_max_ = 0.20 e Å^−^^3^
155 parameters	Δρ_min_ = −0.34 e Å^−^^3^
0 restraints	Absolute structure: Flack (1983), 834 Friedel pairs
Primary atom site location: structure-invariant direct methods	Flack parameter: 0.016 (9)

### Special details

**Table:**

Geometry. All e.s.d.'s (except the e.s.d. in the dihedral angle between two l.s. planes) are estimated using the full covariance matrix. The cell e.s.d.'s are taken into account individually in the estimation of e.s.d.'s in distances, angles and torsion angles; correlations between e.s.d.'s in cell parameters are only used when they are defined by crystal symmetry. An approximate (isotropic) treatment of cell e.s.d.'s is used for estimating e.s.d.'s involving l.s. planes.
Refinement. Refinement of *F*^2^ against ALL reflections. The weighted *R*-factor *wR* and goodness of fit *S* are based on *F*^2^, conventional *R*-factors *R* are based on *F*, with *F* set to zero for negative *F*^2^. The threshold expression of *F*^2^ > σ(*F*^2^) is used only for calculating *R*-factors(gt) *etc*. and is not relevant to the choice of reflections for refinement. *R*-factors based on *F*^2^ are statistically about twice as large as those based on *F*, and *R*- factors based on ALL data will be even larger.

### Fractional atomic coordinates and isotropic or equivalent isotropic displacement parameters (Å^2^)

**Table:**

	*x*	*y*	*z*	*U*_iso_*/*U*_eq_	
Br1	0.27277 (5)	−0.100786 (17)	0.715735 (13)	0.03335 (9)	
N1	0.5109 (4)	0.10657 (15)	0.81851 (10)	0.0218 (4)	
N2	0.4796 (4)	0.08084 (15)	0.94956 (10)	0.0263 (5)	
H2C	0.5395	0.0505	0.9904	0.032*	
C1	0.3861 (5)	0.04049 (18)	0.68858 (12)	0.0225 (5)	
H1A	0.2276	0.0878	0.6937	0.027*	
C2	0.6127 (5)	0.07596 (18)	0.74317 (13)	0.0234 (5)	
H2A	0.7521	0.0206	0.7484	0.028*	
C3	0.6883 (5)	0.15620 (18)	0.62383 (11)	0.0262 (5)	
H3A	0.8688	0.1469	0.5994	0.031*	
C4	0.5166 (5)	0.0564 (2)	0.61047 (12)	0.0258 (6)	
H4A	0.3762	0.0675	0.5708	0.031*	
C5	0.5620 (5)	0.2564 (2)	0.59440 (14)	0.0354 (6)	
H5A	0.6704	0.3160	0.6115	0.042*	
H5B	0.5616	0.2560	0.5388	0.042*	
C6	0.6060 (5)	0.05312 (19)	0.88240 (13)	0.0241 (5)	
C7	0.2656 (5)	0.15228 (17)	0.95953 (11)	0.0257 (5)	
C8	0.1829 (5)	0.20597 (17)	0.89049 (12)	0.0244 (5)	
H8A	0.0460	0.2571	0.8921	0.029*	
C9	0.3045 (5)	0.18165 (17)	0.82441 (12)	0.0243 (5)	
H9A	0.2486	0.2166	0.7802	0.029*	
O1	0.7292 (4)	0.16509 (12)	0.70608 (7)	0.0290 (4)	
O2	0.2882 (4)	0.26569 (14)	0.62192 (11)	0.0494 (5)	
H2B	0.2448	0.3279	0.6236	0.074*	
O3	0.7002 (4)	−0.02551 (13)	0.58996 (8)	0.0339 (4)	
H3B	0.6137	−0.0730	0.5692	0.051*	
O4	0.7873 (4)	−0.01293 (12)	0.88008 (8)	0.0332 (4)	
O5	0.1654 (4)	0.16566 (13)	1.02337 (8)	0.0351 (4)	

### Atomic displacement parameters (Å^2^)

**Table:**

	*U*^11^	*U*^22^	*U*^33^	*U*^12^	*U*^13^	*U*^23^
Br1	0.03955 (14)	0.02863 (14)	0.03187 (14)	−0.00614 (13)	0.00305 (12)	−0.00212 (10)
N1	0.0252 (9)	0.0242 (11)	0.0162 (9)	0.0052 (10)	−0.0018 (8)	−0.0013 (9)
N2	0.0350 (11)	0.0281 (12)	0.0157 (10)	0.0027 (10)	−0.0039 (9)	0.0051 (9)
C1	0.0248 (12)	0.0197 (12)	0.0229 (12)	0.0001 (10)	0.0001 (9)	−0.0030 (10)
C2	0.0210 (11)	0.0240 (13)	0.0252 (12)	−0.0011 (10)	0.0022 (10)	−0.0032 (10)
C3	0.0255 (13)	0.0352 (14)	0.0178 (11)	−0.0011 (12)	0.0034 (10)	−0.0009 (10)
C4	0.0246 (12)	0.0309 (14)	0.0218 (12)	0.0025 (12)	−0.0003 (10)	−0.0023 (11)
C5	0.0363 (15)	0.0342 (15)	0.0357 (15)	−0.0072 (13)	−0.0011 (12)	0.0064 (13)
C6	0.0268 (13)	0.0207 (13)	0.0247 (13)	−0.0053 (12)	−0.0044 (10)	0.0017 (11)
C7	0.0277 (13)	0.0260 (12)	0.0235 (11)	−0.0059 (12)	−0.0005 (12)	−0.0015 (9)
C8	0.0265 (13)	0.0241 (12)	0.0225 (12)	0.0026 (11)	−0.0018 (10)	−0.0030 (10)
C9	0.0251 (13)	0.0232 (12)	0.0245 (12)	−0.0002 (11)	−0.0046 (10)	0.0008 (10)
O1	0.0332 (9)	0.0331 (9)	0.0208 (7)	−0.0096 (9)	0.0003 (9)	0.0002 (6)
O2	0.0309 (11)	0.0294 (9)	0.0879 (14)	−0.0012 (10)	0.0003 (11)	0.0049 (9)
O3	0.0382 (10)	0.0327 (9)	0.0309 (9)	0.0040 (9)	0.0079 (8)	−0.0120 (7)
O4	0.0367 (10)	0.0306 (9)	0.0323 (9)	0.0106 (10)	−0.0057 (9)	0.0008 (7)
O5	0.0433 (11)	0.0416 (11)	0.0203 (8)	0.0005 (9)	0.0073 (8)	0.0001 (8)

### Geometric parameters (Å, °)

**Table:**

Br1—C1	1.938 (2)	C3—H3A	0.9800
N1—C6	1.384 (3)	C4—O3	1.416 (3)
N1—C9	1.387 (3)	C4—H4A	0.9800
N1—C2	1.456 (3)	C5—O2	1.416 (3)
N2—C6	1.368 (3)	C5—H5A	0.9700
N2—C7	1.390 (3)	C5—H5B	0.9700
N2—H2C	0.8600	C6—O4	1.216 (3)
C1—C4	1.515 (3)	C7—O5	1.226 (2)
C1—C2	1.522 (3)	C7—C8	1.441 (3)
C1—H1A	0.9800	C8—C9	1.331 (3)
C2—O1	1.422 (3)	C8—H8A	0.9300
C2—H2A	0.9800	C9—H9A	0.9300
C3—O1	1.452 (2)	O2—H2B	0.8200
C3—C5	1.504 (4)	O3—H3B	0.8200
C3—C4	1.536 (3)		
			
C6—N1—C9	121.24 (18)	C1—C4—C3	101.55 (17)
C6—N1—C2	118.83 (18)	O3—C4—H4A	111.3
C9—N1—C2	119.68 (17)	C1—C4—H4A	111.3
C6—N2—C7	127.57 (18)	C3—C4—H4A	111.3
C6—N2—H2C	116.2	O2—C5—C3	109.7 (2)
C7—N2—H2C	116.2	O2—C5—H5A	109.7
C4—C1—C2	102.81 (19)	C3—C5—H5A	109.7
C4—C1—Br1	117.50 (16)	O2—C5—H5B	109.7
C2—C1—Br1	109.06 (15)	C3—C5—H5B	109.7
C4—C1—H1A	109.0	H5A—C5—H5B	108.2
C2—C1—H1A	109.0	O4—C6—N2	122.0 (2)
Br1—C1—H1A	109.0	O4—C6—N1	123.6 (2)
O1—C2—N1	109.36 (18)	N2—C6—N1	114.4 (2)
O1—C2—C1	103.78 (18)	O5—C7—N2	119.96 (19)
N1—C2—C1	113.54 (18)	O5—C7—C8	125.7 (2)
O1—C2—H2A	110.0	N2—C7—C8	114.38 (18)
N1—C2—H2A	110.0	C9—C8—C7	119.4 (2)
C1—C2—H2A	110.0	C9—C8—H8A	120.3
O1—C3—C5	109.06 (19)	C7—C8—H8A	120.3
O1—C3—C4	106.75 (17)	C8—C9—N1	122.9 (2)
C5—C3—C4	115.4 (2)	C8—C9—H9A	118.5
O1—C3—H3A	108.5	N1—C9—H9A	118.5
C5—C3—H3A	108.5	C2—O1—C3	109.45 (16)
C4—C3—H3A	108.5	C5—O2—H2B	109.5
O3—C4—C1	112.99 (19)	C4—O3—H3B	109.5
O3—C4—C3	107.85 (18)		

### Hydrogen-bond geometry (Å, °)

**Table:**

*D*—H···*A*	*D*—H	H···*A*	*D*···*A*	*D*—H···*A*
O2—H2B···O4^i^	0.82	2.03	2.841 (2)	169
N2—H2C···O3^ii^	0.86	2.17	2.983 (2)	158
O3—H3B···O5^iii^	0.82	1.96	2.769 (2)	167

Symmetry codes: (i) −*x*+1, *y*+1/2, −*z*+3/2; (ii) −*x*+3/2, −*y*, *z*+1/2; (iii) −*x*+1/2, −*y*, *z*−1/2.
